# Pulmonary Hypertension: Current Diagnosis, Approach and Treatment at the Dawn of the New European Guidelines

**DOI:** 10.3390/jcm11195804

**Published:** 2022-09-30

**Authors:** Stylianos E. Orfanos, George Giannakoulas

**Affiliations:** 1 1st Department of Critical Care and Pulmonary Services, National and Kapodistrian University of Athens Medical School, Pulmonary Hypertension Center, Evangelismos Hospital, 10676 Athens, Greece; 2 Cardiology Department, AHEPA University Hospital, Aristotle University of Thessaloniki, 54637 Thessaloniki, Greece

A turning point in the field of pulmonary hypertension (PH) is the most recent publication of the new European Guidelines for the diagnosis and treatment of pulmonary hypertension, a collaboration between the European Society of Cardiology and the European Respiratory Society [1]. These Guidelines contain many important novelties, including new PH and pulmonary arterial hypertension (PAH) definitions, mainly related to the mean pulmonary arterial pressure (mPAP) and pulmonary vascular resistance (PVR) thresholds, which are now lowered to 20 mmHg and 2 Wood Units, respectively. Important changes are, in addition, introduced in many sections including risk stratification, diagnostic algorithm, screening, and treatment. An important issue is, among others, the reintroduction of exercise-induced PH [1].

It should be noted that the recently published Special Issue of the *Journal of Clinical Medicine* on Pulmonary Hypertension, Current Diagnosis, Approach and Treatment, was completed just before the publication of the current European Guidelines. As a matter of fact, the very last article—a commentary on the PVR paradox and the importance of pulmonary capillary recruitment in PAH treatment [2]—was published almost simultaneously with the Guidelines. Although the articles of the Special Issue are not in conflict with the new Guidelines, readers should keep in mind that these articles were written prior to the latter and, thus, may not entirely reflect the new material therein.

The Special Issue contains six research articles, five reviews, a comment on a publication and one commentary. Two research articles are related to prognostication in PAH. In the first, a retrospective study on 62 PAH patients, the prognostic value of several biochemical, echocardiographic, and hemodynamic indices during the initial right heart catheterization (RHC), at the time of PAH diagnosis were examined. Patients were followed for 5 years, while death or need for lung transplantation were used as primary endpoints [3]. The authors report that from all indices analyzed, two independent predictors were found to foresee the defined primary endpoints, namely (i) the stroke volume index (SVI) during reversibility test with inhaled nitric oxide (iNO) at RHC, and (ii) the arterial oxygen saturation. The second article by Qaderi et al. [4] studied right heart echocardiographic parameters in conjunction with other functional and laboratory parameters in a group of 254 PAH patients, in an effort to identify those related to poor outcome. An additional aim was the potential development of a non-invasive, echocardiographic risk score. Using Cox regression models, the authors studied an extended echocardiographic model versus a conventional one and tested the related strengths in predicting all-cause death or lung transplantation in a median follow-up time of 4.2 years. The authors conclude that the former model proved stronger that the latter. The newly proposed risk score included functional, exercise-related, biochemical and echocardiographic indices, pending independent external validation [4].

A third publication, a review, is also examining the prognostic utility of echocardiography [5]. The authors emphasize the importance of right ventricular (RV) function in PAH risk stratification; echocardiography plays a crucial role in identifying the underlying pathological patterns of RV dysfunction. The authors prominently show the importance of echocardiography at diagnosis and during the disease process; they also point out that, despite the use of numerous echocardiographic parameters and their previously shown prognostic values, their use in patients’ risk assessment is rather limited and should be further studied. Of note, latest European Guidelines; the latter, in compliance with this review [5] do emphasize the importance of echocardiography in diagnosis, risk assessing, and patients’ follow up.

The important role of echocardiography in PH and more particularly in PAH is further revealed by the fact that two additional publications and a comment are also added in this group of papers. Rallidis et al., studied the role of stress echocardiography with the administration of low-dose dobutamine in patients suffering from systemic sclerosis (SSc), in whom resting echocardiograms were not diagnostic for PH [6]. All patients underwent RHC prior to heart echocardiograms and patients with and without PH were compared. Several echocardiographic indices were different between the dobutamine stress echocardiographic studies of the two groups, with tricuspid regurgitation (TR) velocity showing high sensitivity, specificity and accuracy in detecting PAH. These findings suggest that low dose dobutamine stress echocardiography may have an important role in the early diagnosis of PAH in SSc patients [6]. A comprehensive review on the role of echocardiography in PAH that includes technical information on the new techniques in use, and emphasizes the need for testing, standardization and precise methodology offers useful detailed information on the technique [7]. The related comment by Manzi et al., that followed, emphasizes the need of including in the echocardiographic testing indices reflecting the pathophysiological changes that occur in the process of the disease and which, most importantly, would test the effect of specific PAH therapies [8].

An important issue, that is a comprehensive approach of the hemoptysis occurring in PAH patients secondary to congenital heart disease is presented by Baroutidou et al. [9]. It appears that the pulmonary vascular pathobiology that accompanies this disorder predisposes patients to hemoptysis. The authors report that despite the major advances of the recent past, hemoptysis is always associated with increased morbidity and mortality in these high-risk patients. In addition to the related pathophysiology, diagnostic approaches, therapeutic options and future directions are also discussed [9].

Two reviews that address rather rarely discussed issues follow: A systematic review and meta-analysis on nailfold capillaroscopy on SSc subjects with and without PAH revealed that SSc patients with PAH had overall worse capillaroscopic findings compared to the latter subjects [10]. This suggests a microvascular systemic involvement in PAH and might assist the clinician to earlier identify PAH in this setting. The second review assesses the direct or indirect role of thyroid diseases, obesity, diabetes mellitus, and estrogens in PH and PAH [11]. Importantly, this review comments on potential pregnancy in PAH. These comments should be viewed in conjunction with the recommendations for women of childbearing potential given in the European Guidelines [1,11].

The Special Issue also includes three important publications related to a national referral center in Spain and the Hellenic national registry, all related to chronic thromboembolic pulmonary hypertension (CTEPH): Cruz-Utrilla et al., report on sex-related differences in CTEPH; data are provided via the Spanish registry REHAP, but are related to one center only. In total, 453 patients were evaluated between 2007–2019, revealing among others that female patients had worse functional, exercise-related, and PVR profiles than men, but more pulmonary endarterectomy (PEA) surgical procedures were performed in the latter [12]. The authors report a better survival only in women between 2014–2019, that might be related to the introduction of balloon pulmonary angioplasty (BPA) and the improvement in the performed PEAs [12]. The remaining two articles present data from the Hellenic nationwide pulmonary hypertension registry (HOPE). Demerouti et al., provide detailed data on Greek CTEPH patients between 2015 and 2019. Ιn total, 98 patients were enrolled; differences between operable and non-operable CTEPH patients in real world are presented [13]. Finally, Karyofyllis et al., provide data from 180 BPA procedures performed in Greece. Improvements in mPAP, PVR, and NT-proBNP were noticed, accompanied by modest improvement in cardiac index. BPAs provide thus another treatment option to the eligible patients in Greece [14].

Last but not least, in the Special Issue is the commentary by Langleben et al., on the PVR paradox and the significance of restoring pulmonary capillary recruitment for a true therapeutic intervention. Why PVR paradox? It is because when PVR decreases due to an increase in cardiac output (CO), as a result of vasodilator therapies, it is not related to precapillary de-modelling; the latter would allow true restoration of capillary recruitment with increasing CO, such as in exercise, i.e., true restoration of pulmonary microvascular physiology [2]. Due to the aforementioned phenomenon, until now there have been treatments for PAH, but no cure. The novel anti-proliferative therapies, if successful, should point towards the direction of direct decrease in afterload. Testing such therapies not only at rest but also under exercise should unveil the true effectiveness of potential antiproliferative medications.

The Special Issue of the *Journal of Clinical Medicine* on Pulmonary Hypertension, Current Diagnosis, Approach and Treatment was completed at the dawn of the new European Guidelines. The authors of this Editorial believe that it has added valued information to our knowledge on the field of PH and PAH. This is a field under constant development that should hopefully lead to the cure of this devastating disease in the non-distant future.
